# Citizen‐Science Camera Trap Data Reveal Large‐Scale Activity Patterns of the Bobcat (*Lynx rufus*) Across Mexican Ecosystems

**DOI:** 10.1002/ece3.73917

**Published:** 2026-07-01

**Authors:** Luis A. Alanis‐Hernández, Amayrani E. Trejo‐Montero, Gerardo Sánchez‐Rojas, Jaime Manuel Calderón‐Patrón, Mario C. Lavariega, Juan Pablo Esparza‐Carlos, Luis Ignacio Íñiguez‐Dávalos

**Affiliations:** ^1^ Departamento de Ecología y Recursos Naturales, Centro Universitario de la Costa Sur Universidad de Guadalajara Autlán de Navarro Jalisco México; ^2^ Predio Intensivo de Manejo de Vida Silvestre X‐Plora Reptilia Metztitlán Hidalgo México; ^3^ Laboratorio de Conservación Biológica, Centro de Investigaciones Biológicas (CIB) Instituto de Ciencias Básicas e Ingeniería de la Universidad Autónoma del Estado de Hidalgo Mineral de la Reforma Hidalgo México; ^4^ Laboratorio de Biodiversidad, Escuela de Ciencias Universidad Autónoma Benito Juárez de Oaxaca Oaxaca de Juárez México; ^5^ Centro Interdisciplinario de Investigación para el Desarrollo Integral Regional, Unidad Oaxaca Instituto Politécnico Nacional Santa Cruz Xoxocotlán Oaxaca México

**Keywords:** behavioral plasticity, circular statistics, mesocarnivore ecology, temporal niche, ecología de mesocarnívoros, estadística circular, nicho temporal, plasticidad conductual

## Abstract

Daily activity patterns represent a key behavioral component of carnivores, shaped by resource availability, environmental conditions, intra‐ and interspecific interactions, and human disturbance. The bobcat ( *Lynx rufus* ) is a widely distributed mesocarnivore across North America; however, its activity patterns in Mexico have been scarcely evaluated at broad spatial scales. In this study, we analyzed the potential of citizen science records to characterize bobcat activity patterns at a national scale in Mexico. We analyzed 821 independent photographic records collected between 2005 and 2025 from the iNaturalist platform, distributed across 25 states and encompassing both relatively conserved environments and human‐modified landscapes. Recorded were classified into six main vegetation types: Temperate forests, Grasslands, Shrublands, Tropical forests, Riparian vegetation, and Agricultural‐modified areas. We evaluated activity patterns using kernel density functions, overlap coefficients, and circular statistics. At the national scale, the bobcat exhibited a general cathemeral activity pattern, with records distributed throughout the 24‐h cycle but concentrated during crepuscular and nocturnal periods. This pattern remained broadly consistent across vegetation types, although the position and intensity of activity peaks varied among habitats. In particular, Temperate forests showed a higher proportion of diurnal records, suggesting a more even distribution of activity throughout the day compared with other habitats. Overall, these results highlight the temporal plasticity of the bobcat across heterogeneous environments and demonstrate the potential of citizen science datasets for investigating large‐scale behavioral patterns in widely distributed carnivores.

## Graphical abstract

We evaluated whether citizen science–derived camera‐trap records can be used to characterize large‐scale activity patterns of a widely distributed mesocarnivore across heterogeneous landscapes. Using 821 independent iNaturalist records, we quantified activity across six major vegetation types in Mexico with kernel density estimation, circular statistics, and overlap analyses. The bobcat exhibited a general cathemeral pattern with crepuscular–nocturnal peaks that remained broadly consistent among vegetation types, although temperate forests showed a higher proportion of diurnal activity.

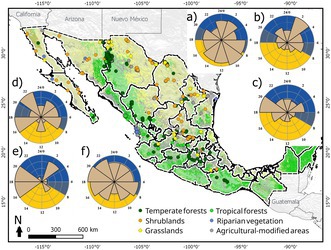

## RESUMEN

Los patrones de actividad diaria representan un componente conductual clave de los carnívoros, modulados por la disponibilidad de recursos, las condiciones ambientales, las interacciones intra e interespecíficas y la perturbación humana. El gato montés ( *Lynx rufus* ) es un mesocarnívoro ampliamente distribuido en Norteamérica; sin embargo, sus patrones de actividad en México han sido escasamente evaluados a gran escala. En este estudio, analizamos el potencial de los registros de ciencia ciudadana para caracterizar los patrones de actividad del gato montés a escala nacional en México. Analizamos 821 registros fotográficos independientes recopilados entre 2005 y 2025 de la plataforma iNaturalist, distribuidos en 25 estados de México y que abarcan tanto entornos relativamente conservados como paisajes modificados por el hombre. Los registros se analizaron y se clasificaron en seis tipos principales de vegetación: Bosques templados, Pastizales, Matorrales, Bosques tropicales, Vegetación ribereña y Áreas agrícolas modificadas. Evaluamos los patrones de actividad utilizando funciones de densidad de Kernel, coeficientes de traslape y estadística circular. A escala nacional, el gato montés presentó un patrón general de actividad catemeral, con registros distribuidos a lo largo del ciclo de 24 horas, pero con mayor concentración durante los periodos crepusculares y nocturnos. Este patrón fue ampliamente consistente entre tipos de vegetación, aunque la posición e intensidad de los picos de actividad variaron entre hábitats. En particular, los bosques templados presentaron una mayor proporción de registros diurnos, lo que sugiere una distribución más uniforme de la actividad a lo largo del día en comparación con el resto de los hábitats. En conjunto, estos resultados resaltan la plasticidad temporal del gato montés y demuestran el potencial de los conjuntos de datos de ciencia ciudadana para analizar patrones de comportamiento a gran escala en carnívoros ampliamente distribuidos.

## 1 Introduction

In recent decades, citizen science platforms have become valuable tools for ecological research by enabling the collection of large volumes of information generated by volunteer observers across broad spatial and temporal scales (Hsing et al. 2022; Green et al. 2023). These initiatives have contributed significantly to biodiversity monitoring by providing data that complement traditional approaches (Dickinson et al. 2010; Bonney et al. 2016; McKinley et al. 2017; Cuervo‐Robayo et al. 2026). Among these platforms, iNaturalist has become one of the most important repositories of biological observations worldwide by integrating georeferenced records with verifiable photographic evidence. These datasets have been used to document species distributions, phenological changes, ecological interactions, and spatial patterns of biodiversity (Stephenson and Stengel 2020; Weissgold 2024; Herrera et al. 2025).

In addition to their usefulness for distributional and diversity studies, photographic records generated by camera traps and shared on citizen science platforms offer growing potential for analyzing wildlife behavioral patterns, including daily activity patterns (Hsing et al. 2022). These analyses rely on timestamps associated with photographs, allowing researchers to infer activity of different species (O'Connell et al. 2011; Rowcliffe et al. 2014; Burton et al. 2015). Unlike traditional camera‐trapping studies, which are often limited to specific areas or regional scales, citizen science data can encompass extensive regions and diverse habitat types with different vegetation characteristics, providing a unique opportunity to evaluate ecological patterns at broad biogeographic scales (Villafañe‐Trujillo et al. 2021; Vallejo‐Vargas et al. 2022).

Carnivore behavior is influenced by multiple interacting biotic and abiotic factors, including prey availability (López‐Vidal et al. 2014; Annear et al. 2025), intra‐ and interspecific competition (Shores et al. 2019; Durán‐Antonio et al. 2020; Vilella et al. 2020), as well as habitat characteristics, seasonality, human activities, and the degree of ecosystem disturbance (Lendrum et al. 2017; Azevedo et al. 2018; Guerisoli et al. 2019; Pérez‐Irineo et al. 2021; Granados et al. 2024; Guzmán‐Aguayo et al. 2025). These factors play a key role in shaping predator temporal activity, influencing foraging strategies, niche partitioning with other species, and interactions with human‐modified environments (Lendrum et al. 2017; Granados et al. 2024). Among North American mesocarnivores, the bobcat ( *Lynx rufus* ) is an important model for evaluating such behavioral flexibility because of its wide geographic distribution and ability to occupy environmentally heterogeneous landscapes.

The bobcat, a resilient mesocarnivore widely distributed across North America (Hall 1981), is characterized by a remarkable ability to adapt to different environments, including highly modified landscapes (Bárcenas and Medellín 2021; Espinosa‐Flores et al. 2022). It is a generalist predator whose diet includes a wide variety of prey, primarily lagomorphs and rodents (Tewes et al. 2002; Draper et al. 2022; Landry et al. 2022; Alanis‐Hernández et al. 2023; Alanis‐Hernández et al. 2026). Due to this ecological flexibility, the species plays an important ecological role in regulating prey populations and shaping mesocarnivore community dynamics, particularly through competition and niche segregation processes (Crooks and Soulé 1999; Ritchie and Johnson 2009).

The temporal behavior of *L. rufus* has been described as particularly flexible, with activity patterns that may be predominantly nocturnal (Harris et al. 2015; Hubbard et al. 2022; Procko et al. 2023), crepuscular (Chamberlain et al. 1998; Rockhill et al. 2013; Tigas et al. 2002; Wang et al. 2015), or even cathemeral, with activity distributed throughout both day and night (O'Connor and Rittenhouse 2017; Serna‐Lagunes et al. 2019). Across several localities within its northern distribution, numerous studies have documented this behavioral variability and its relationship with environmental factors, prey availability, and human pressure (Lendrum et al. 2017; Shores et al. 2019; Prat‐Guitart et al. 2020; Murphy et al. 2021; Rodriguez et al. 2021; Hubbard et al. 2022; Procko et al. 2023; Granados et al. 2024).

In Mexico, which represents the southern portion of the species' distribution, knowledge of bobcat activity patterns remains limited and is based on relatively few regional studies (Elizalde‐Arellano et al. 2012; Flores‐Morales et al. 2019; Serna‐Lagunes et al. 2019; Durán‐Antonio et al. 2020; Andrade‐Ponce et al. 2022; Lavariega et al. 2022). This is particularly relevant considering that the country harbors a high diversity of ecosystems within the species' range (> 70% of the states), including temperate forests, arid and semiarid environments, riparian vegetation, and landscapes heavily transformed by human activities (Espinosa‐Flores and López‐González 2017; Espinosa‐Flores et al. 2022). This environmental heterogeneity may generate important variation in bobcat temporal activity associated with differences in habitat structure and levels of anthropogenic disturbance. However, to date, no national‐scale analysis has described activity patterns across multiple vegetation types due to the lack of data spanning its distribution.

Despite the growing volume of data available on citizen science platforms, their usefulness for analyzing carnivore activity patterns at broad spatial scales remains poorly evaluated, particularly in megadiverse countries such as Mexico. Most previous studies on bobcat activity in Mexico have been restricted to local or regional camera‐trap surveys, limiting understanding of how activity patterns vary across heterogeneous environments and vegetation types at a national scale. Citizen‐science records provide an opportunity to address these large‐scale ecological questions using geographically extensive datasets that would be difficult to obtain through traditional monitoring programs alone (Mendoza et al. 2022).

The objectives of this study were: (1) to evaluate the potential of records derived from the citizen science platform iNaturalist for analyzing bobcat activity patterns at broad spatial scales, (2) to describe the general activity pattern in Mexico, and (3) to compare temporal activity patterns among the main vegetation types present in the country.

We expected the bobcat to exhibit a nonuniform activity pattern, but rather one predominantly nocturnal–crepuscular (Chamberlain et al. 1998; Tigas et al. 2002; Rockhill et al. 2013; Harris et al. 2015; Wang et al. 2015; Hubbard et al. 2022; Procko et al. 2023). We also hypothesized that temporal activity would vary among vegetation types, possibly associated with habitat structure, prey availability, and the degree of anthropogenic disturbance.

## 2 Materials and Methods

### 2.1 Study Area

**FIGURE 1:**
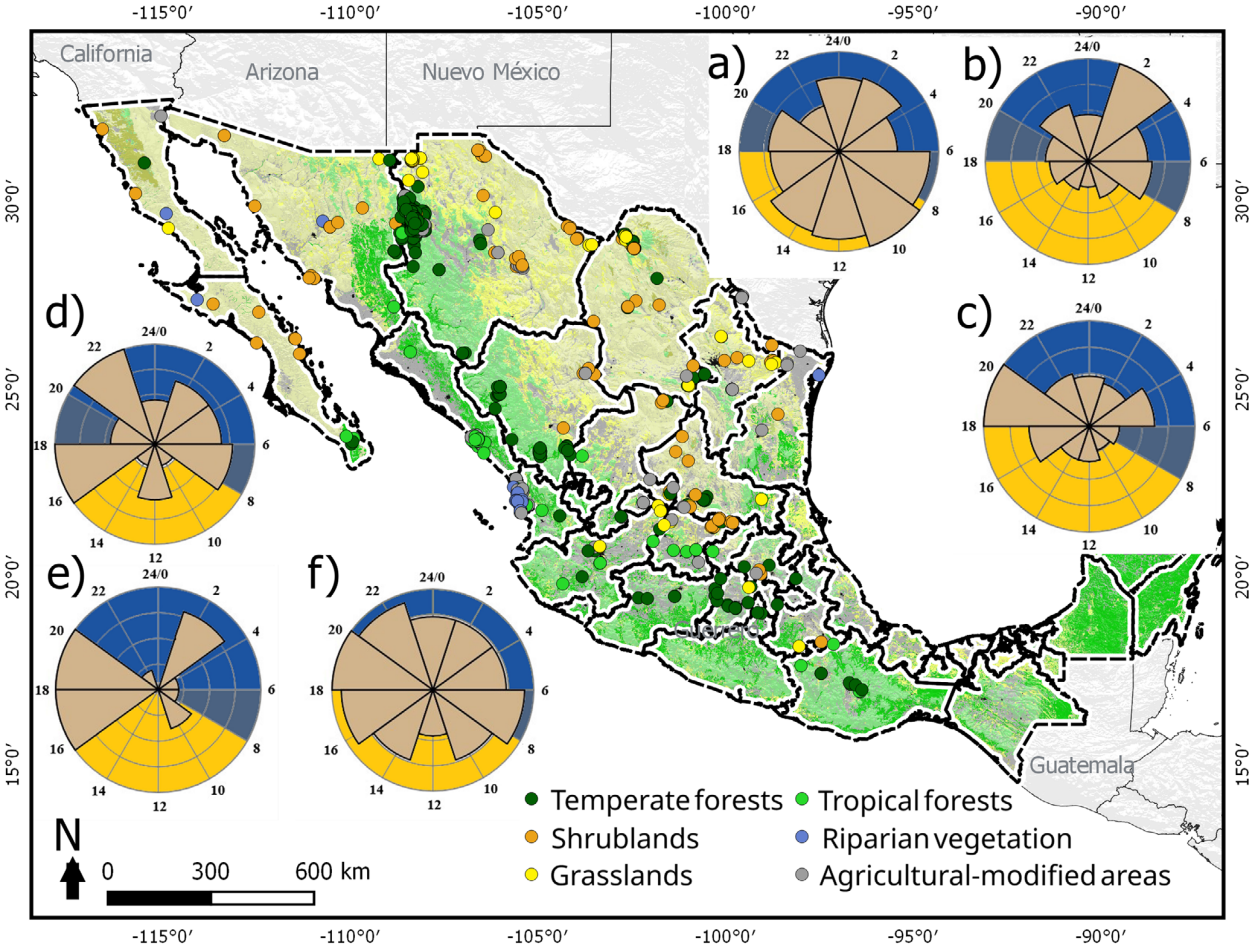
Activity patterns of the bobcat ( *Lynx rufus* ) across vegetation types and agricultural‐modified areas in Mexico based on citizen science records from iNaturalist (*n* = 821). (a) Temperate forests, (b) Grasslands, (c) Shrublands, (d) Tropical forests, (e) Riparian vegetation, and (f) Agricultural‐modified areas. The crystal‐blue shaded area corresponds to nighttime, the steel‐blue area represents sunrise and sunset, and the yellow area corresponds to daytime. Numbers around the circle indicate hours of the day, resembling a clock. The number of records is shown vertically and represented in the graph as beige bars.

We analyzed at a national scale, considering all georeferenced bobcat records within Mexican territory (Figure 1). Mexico exhibits marked environmental heterogeneity characterized by latitudinal, altitudinal, and climatic gradients that include arid zones, temperate forests, tropical forests, and environments highly modified by human activities (Rzedowski 2006; INEGI 2021; CONABIO 2025).

### 2.2 Data Source and Record Compilation

We compiled all available “Research Grade” records for the species from the citizen science platform iNaturalist (https://mexico.inaturalist.org/) corresponding to the period 2005–2025. The search used the keywords “*Lynx*” and “*rufus*.” The preliminary database included the following information associated with each record: record ID, author, locality, date, geographic coordinates, image URL, common name, and scientific name.

### 2.3 Data Filtering and Record Selection

Because records derived from citizen science platforms may show oversampling associated with the spatial distribution of sampling effort, we applied selection criteria to reduce their potential effect on the analyses. We restricted records to those obtained with camera‐trap devices, thereby reducing the possibility of changes in individual activity caused by observer presence (Caravaggi et al. 2017). We also verified the agreement between the recorded time and the lighting conditions observable in photographs (e.g., daylight, low‐light crepuscular conditions, or nighttime illumination), as well as the spatial consistency of the geographic coordinates associated with each record. This procedure allowed us to identify and exclude records with incorrect georeferencing or evident temporal and spatial inconsistencies (e.g., records located outside continental territory). These measures improved the reliability of activity analyses derived from opportunistic records, as noted in previous studies using citizen science data (Dickinson et al. 2010; Isaac et al. 2014; Callaghan et al. 2019).

### 2.4 Database Construction

To reduce pseudoreplication, only independent records were included in the analyses. Consecutive records obtained from the same camera location were considered independent only when separated by at least 30 min, a threshold commonly used in camera‐trap studies of mammalian activity patterns. When multiple photographs of bobcats were recorded within a shorter interval, only the first detection event was retained from the downloaded iNaturalist dataset. This criterion was based on previous camera‐trap activity studies that recommended using independent detections to avoid overrepresentation of records associated with the same individual (O'Brien et al. 2003; Ridout and Linkie 2009; Linkie and Ridout 2011; Esparza‐Carlos et al. 2025).

The final database comprised 821 independent camera‐trap records. For each record, we compiled the following information: ID, Year, State, Latitude, Longitude, Date (day/month/year), Julian day, Time (h:min:s, 24 h), Solar time, Radians, and URL (Dataset S1).

### 2.5 Time Standardization and Conversion to Solar Time

We standardized record times in R version 4.5.1 (R Core Team 2026), following the procedure described by Lara‐Díaz et al. (2018). The process included three steps: (1) conversion of the local time recorded in the photographs to solar time using the package solaR (Perpiñán‐Lamigueiro 2012); (2) calculation of average sunrise and sunset times for each record using the package RAtmosphere (Biavati 2015); and (3) obtaining Julian dates using the online tool from the United States Naval Observatory (USNO 2015), which are required for calculating astronomical variables in RAtmosphere. Solar times were subsequently converted to radians for circular statistical analyses. This procedure standardized records and allowed comparison of activity patterns across different regions of the country.

### 2.6 Vegetation Classification of Records Locations

The vegetation type associated with each record was determined using QGIS version 2.28 (QGIS Development Team 2022) and the Serie VII land‐use and vegetation map from the Instituto Nacional de Estadística y Geografía (Instituto Nacional de Estadística y Geografía (INEGI) 2021). Given that records spanned a wide temporal range (2005–2025), potential temporal inconsistencies in vegetation assignment were evaluated by comparing coverages reported in Serie III (2002–2005) and Serie VII (2017–2021) from INEGI. This comparison did not reveal substantial changes in vegetation types at the scale of analysis used, indicating that Serie VII provides an adequate and consistent representation of prevailing conditions within the study area.

Because Serie VII includes more than 50 specific categories, we grouped them into six main vegetation types widely used in ecological studies in Mexico (Rzedowski 2006; Instituto Nacional de Estadística y Geografía (INEGI) 2021; Comisión Nacional para el Conocimiento y Uso de la Biodiversidad CONABIO 2025): Temperate forests, Grasslands, Shrublands, Tropical forests, Riparian vegetation, and Agricultural‐modified areas. We based this reclassification on physiognomic criteria, which allowed consistent comparisons of bobcat activity patterns among broad vegetation classes. Using aggregated categories also reduces sensitivity to potential fine‐scale changes in vegetation cover over time.

## 3 Activity Pattern Analyses

### 3.1 Circular Statistics

Because temporal data are cyclical, we analyzed activity patterns using circular statistics. We applied two tests: (1) Rayleigh test (R) to evaluate the uniformity of the temporal distribution of records. This test is analogous to a normality test for noncircular data. (2) Watson–Williams test (*U* ^2^) to compare activity patterns among vegetation types and detect statistically significant differences among angular means (Cukor et al. 2024). We conducted analyses using PAST software (Hammer 2016).

### 3.2 Estimation of Daily Activity Pattern

We estimated daily activity patterns using kernel density functions implemented in the package overlap in R (Meredith and Ridout 2018). To estimate overlap between temporal distributions, we used the following estimators: Δ1 (Dhat1) when sample size per category was < 75 records, and Δ4 (Dhat4) when sample size was > 75 records, following recommendations by Ridout and Linkie (2009).

Exploratory seasonal analyses were also conducted to evaluate potential differences between reproductive and nonreproductive periods (Figure S3). Records were grouped into reproductive (February–June) and nonreproductive (July–January) periods based on previously described bobcat reproductive phenology (Crowe 1975; Winegarner and Winegarner 1982). Activity patterns for both periods were estimated using kernel density functions and compared using temporal overlap coefficients. Because records were opportunistically collected and unevenly distributed among years (Figure S1), analyses were conducted using pooled records across all years (Figure S2) and should be interpreted as exploratory.

### 3.3 Classification of Activity Periods

Following Ávila‐Nájera et al. (2016) and Lendrum et al. (2017), we classified records into four activity periods: nocturnal (20:00–6:00 h), diurnal (8:00–18:00 h), crepuscular (6:00–8:00 h and 18:00–20:00 h), and cathemeral (activity distributed throughout day and night).

## 4 Results

### 4.1 Citizen Science–Derived Records

We obtained a total of 821 independent photographic records of the bobcat from the iNaturalist platform corresponding to the period 2005–2025. Records spanned 25 states in Mexico and were classified into six main vegetation types: Temperate forests (53.84%), Shrublands (14.49%), Grasslands (12.30%), Agricultural‐modified areas (10.48%), Tropical forests (6.58%), and Riparian vegetation (2.31%).

### 4.2 General Activity Pattern in Mexico

At the national scale, the bobcat showed activity records distributed throughout the 24‐h cycle, indicating a general cathemeral activity pattern. Overall, 40.32% of records were classified as diurnal, 39.83% as nocturnal, and 19.85% as crepuscular. Despite this broad distribution, kernel density estimates revealed distinct peaks of activity around crepuscular and nocturnal periods (Figure 1).

This general pattern remained consistent across most vegetation types, although riparian vegetation showed a more distinctive temporal distribution and variation occurred in the intensity and position of activity peaks. In general, diurnal activity was relatively low compared with crepuscular and nocturnal periods in nearly all vegetation categories, except in temperate forests, where diurnal records represented (% 46.51) of observations.

Exploratory comparisons between reproductive and nonreproductive periods showed high temporal overlap in bobcat activity patterns (Dhat4 = 0.88; Figure S3), suggesting relatively consistent activity schedules across periods when records were pooled among years.

### 4.3 Daily Activity Patterns by Vegetation Type (Kernel)

**FIGURE 2:**
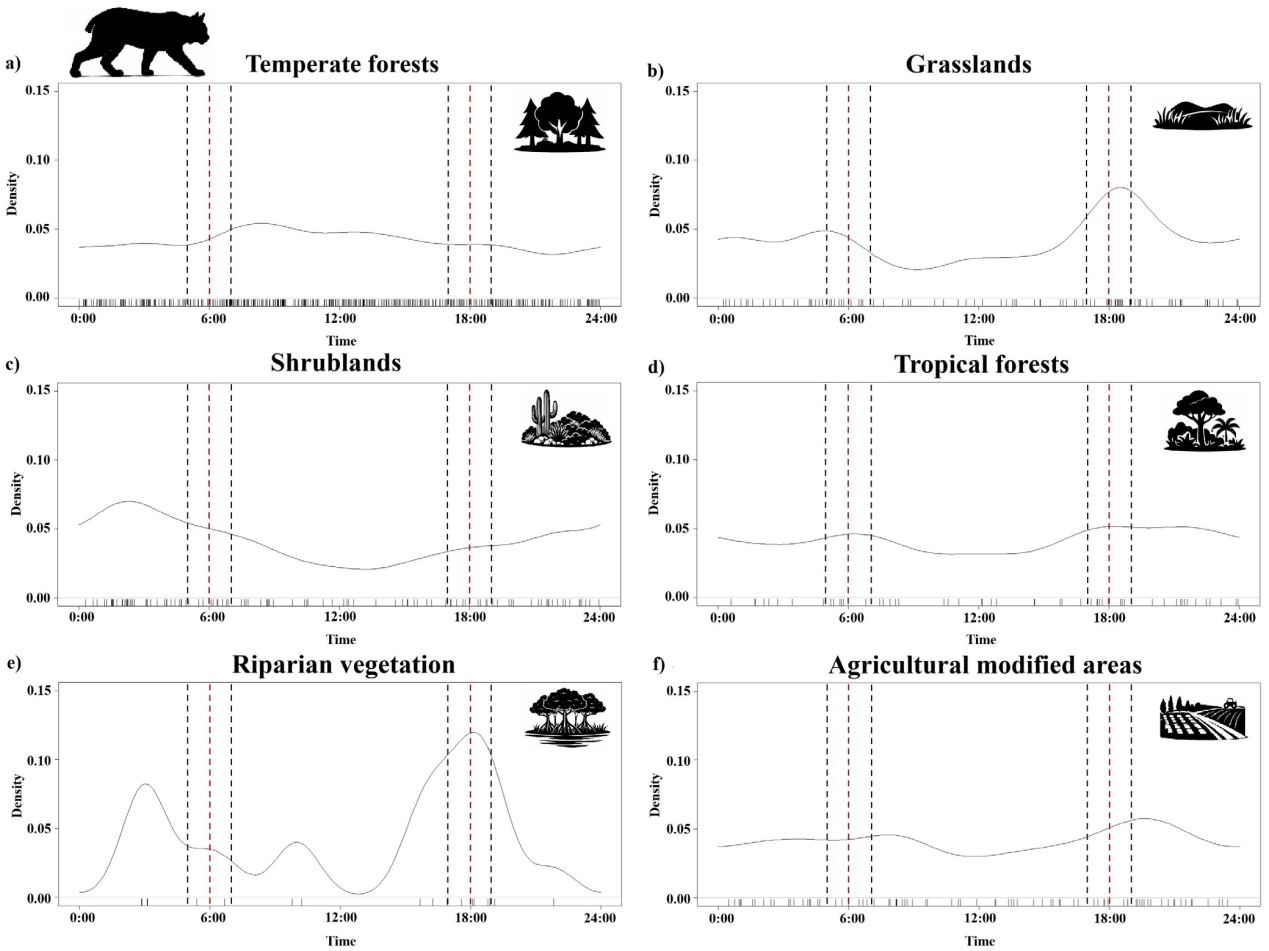
Daily activity patterns of the bobcat ( *Lynx rufus* ) across different vegetation types and agricultural‐modified areas in Mexico. (a) Temperate forests, (b) Grasslands, (c) Shrublands, (d) Tropical forests, (e) Riparian vegetation, and (f) Agricultural‐modified areas. Vertical dashed lines indicate periods corresponding to dawn twilight (±1 h before 06:00) and dusk twilight (±1 h before 18:00).

Kernel density analyses revealed activity distributed throughout the entire daily cycle in all vegetation types, although differences occurred in temporal concentration, and peak activity was observed (Figure 2).

In Temperate forests, bobcat activity was relatively evenly distributed across 24 h, with a moderate proportion of diurnal records and a gradual increase in activity during early morning, reaching the highest density approximately between 07:00–09:00 h.

In Shrublands, Tropical forests, and Agricultural‐modified areas, activity showed moderate increases near sunrise (∼05:30–06:30 h) and sunset (∼18:00–19:30 h), producing relatively broad distributions across the daily cycle. In Grasslands, the pattern was similar but with a greater concentration of activity during the evening crepuscular period, with a more pronounced peak around 18:00–20:00 h.

In contrast, Riparian vegetation showed the most distinctive pattern, characterized by an early nocturnal peak between 02:00 and 04:00 h and a second increase in activity between 17:00 and 19:00 h, producing a more marked bimodal distribution.

### 4.4 Activity Overlap Among Vegetation Types

Activity overlap analysis using the Δ4 (Dhat4) estimator showed generally high overlap coefficients among vegetation types (range: 0.80–0.95; Table 1), indicating broad similarity in bobcat temporal activity patterns. However, comparisons involving Riparian vegetation showed the lowest overlap coefficients (0.65–0.75), suggesting greater differences in temporal pattern in this environment.

**TABLE 1:** Temporal overlap (Δ) of daily activity of the bobcat ( *Lynx rufus* ) among vegetation types.

Types of vegetation	Temperate forests	Grasslands	Shrublands	Tropical forests	Riparian vegetation	Agricultural‐modified areas
Temperate forests		0.8	0.8	0.87	0.65	0.89
Grasslands			0.81	0.9	0.75	0.9
Shrublands				0.87	0.7	0.85
Tropical forests					0.69	0.95
Riparian vegetation						0.7
Agricultural‐modified areas						

Circular statistics analysis using the Watson–Williams test (*U* ^2^) revealed significant differences in mean activity direction among some vegetation types (Table 2), particularly in comparisons involving Temperate forests (*U* ^2^ > 7; *p* < 0.05). In contrast, most remaining comparisons did not show significant differences (*U* ^2^ < 3; *p* > 0.05), indicating relatively similar temporal patterns among those environments.

**TABLE 2:** Pairwise comparisons of bobcat ( *Lynx rufus* ) activity patterns among vegetation types (Watson–Williams *U* ^2^ test).

Vegetation A	Vegetation B	*U* ^2^	*p*
Temperate forests	Grasslands	40.33	< 0.001*
Temperate forests	Shrublands	42.38	< 0.001*
Temperate forests	Agricultural‐modified areas	13.00	< 0.001*
Temperate forests	Tropical forests	11.48	< 0.001*
Temperate forests	Riparian vegetation	7.20	0.008*
Grasslands	Shrublands	12.90	< 0.001*
Grasslands	Agricultural‐modified areas	0.21	0.647
Grasslands	Tropical forests	0.21	0.647
Grasslands	Riparian vegetation	0.63	0.428
Shrublands	Agricultural‐modified areas	3.12	0.079
Shrublands	Tropical forests	2.86	0.093
Shrublands	Riparian vegetation	7.00	0.009*
Tropical forests	Agricultural‐modified areas	0.0001	0.992
Tropical forests	Riparian vegetation	0.81	0.371
Riparian vegetation	Agricultural‐modified areas	0.78	0.379

*Note:* Significant differences are indicated as *.

### 4.5 Temporal Concentration of Activity Pattern (Rayleigh Test)

Results indicated significant clustering of records in some vegetation types, particularly in Temperate forests (*R* = 0.10, *p* = 0.009), Shrublands (*R* = 0.25, *p* < 0.001), and Grasslands (*R* = 0.19, *p* = 0.026), suggesting that activity in these environments is not uniformly distributed throughout the day but instead shows periods with higher frequency of records.

In contrast, Tropical forests (*R* = 0.10, *p* = 0.586), Riparian vegetation (*R* = 0.22, *p* = 0.396), and Agricultural‐modified areas (*R* = 0.07, *p* = 0.646) did not show significant deviations from a uniform distribution, indicating that hourly records in these environments do not cluster around a specific time of day.

## 5 Discussion

### 5.1 Potential and Limitations of Citizen Science for Behavioral Studies

This study demonstrates that records from citizen science platforms, particularly iNaturalist, can represent a valuable source of information for analyzing mammal activity patterns at broad spatial scales. The dataset used in this study enabled us to compile 821 independent camera‐trap records of bobcat distributed across 25 states in Mexico, providing a geographic coverage that would be difficult to achieve with traditional monitoring schemes based exclusively on systematic camera trapping.

Several studies have noted that large volumes of information generated by volunteer observers can complement structured monitoring schemes, particularly for widely distributed or difficult‐to‐detect species (Dickinson et al. 2010; McKinley et al. 2017). In this context, recent evaluations have shown that biodiversity patterns derived from citizen science platforms can be comparable to those obtained using systematic sampling designs. For example, Herrera et al. (2025) found that mammal richness estimates derived from iNaturalist data reflect spatial patterns comparable to those obtained through large‐scale standardized camera‐trap monitoring programs such as the Tropical Ecology Assessment and Monitoring (TEAM) Network (Ahumada et al. 2011), although both approaches capture partially distinct subsets of species.

In addition, national citizen science–based projects have demonstrated their potential to document ecological phenomena at broad scales. Initiatives such as “Mamíferos atropellados en carreteras de México” (iNaturalist 2025) compile large numbers of georeferenced wildlife observations that can identify spatial and temporal patterns of wildlife mortality that would be difficult to detect through conventional monitoring efforts. Although citizen science platforms offer opportunities to compile large volumes of ecological information at broad spatial scales, data generated through these schemes are inherently biases by their opportunistic nature (Dickinson et al. 2010; Isaac et al. 2014; Callaghan et al. 2019). Among the most documented are heterogeneity in sampling effort, concentration of records in accessible areas or near urban centers, and variability in species detectability. In addition, records obtained from citizen‐science platforms represent presence‐only data generated under nonstandardized sampling effort, which limits the possibility of estimating detection probabilities or true activity rates across environments (Fraisl et al. 2022; Geurts et al. 2023; Johnston et al. 2023).

The patterns described in this study should be interpreted as temporal distributions of detections rather than direct estimates of absolute bobcat activity or habitat use. Nevertheless, recent studies have emphasized that large‐scale citizen‐science datasets can still provide robust ecological insights when analytical limitations are explicitly recognized and appropriate filtering procedures are applied (Fraisl et al. 2022; Johnston et al. 2023). In particular, broad‐scale temporal patterns may remain informative even under heterogeneous sampling conditions provided they are interpreted cautiously and supported by sufficiently large sample sizes distributed across multiple regions (Geurts et al. 2023).

In our study, one manifestation of this heterogeneity was the low number of records available for Riparian vegetation, which likely reflects differences in sampling effort among habitat types and limits the robustness of our results and inferences for this specific environment. To reduce some of these limitations, we restricted analyses to research‐grade iNaturalist camera‐trap records with verifiable timestamps, validated temporal and geographic consistency of observations, and applied independence criteria commonly used in camera‐trap studies. While these procedures cannot fully eliminate biases inherent in opportunistic sampling, they enhance the reliability and comparability of temporal activity analyses derived from open‐access datasets. Consequently, the patterns described should be interpreted as temporal distributions of detections rather than as direct estimates of activity rates or habitat use. Despite these limitations, the use of citizen science remains valid for ecological studies and underscores the necessity of rigorous filtering and data‐selection procedures (Fraisl et al. 2022; Johnston et al. 2023). In this sense, integrating records from open platforms with traditional monitoring schemes represents a promising approach for strengthening the study of behavioral ecology in widely distributed species.

### 5.2 General Activity Pattern of the Bobcat in Mexico

Results from this study indicate that the bobcat maintains a general cathemeral activity pattern, characterized by records distributed throughout the 24‐h cycle but with a greater concentration of activity during crepuscular and nocturnal periods. This pattern is consistent with what has been documented for the species across several regions of North America, where bobcats typically show greater activity during periods of low light, particularly around dawn and dusk (Lovallo and Anderson 1996; Chamberlain et al. 1998; Harris et al. 2015; Lendrum et al. 2017; Shores et al. 2019; Procko et al. 2023). Because our dataset included records from both relatively conserved environments (e.g., Temperate forests and riparian vegetation) and human‐modified landscapes (Agricultural‐modified areas), the observed variation in temporal concentration among vegetation types may partially reflect differences in anthropogenic disturbance. Records from Agricultural‐modified areas maintained predominantly crepuscular–nocturnal activity patterns, consistent with behavioral adjustments described for carnivores inhabiting human‐dominated landscapes (Lendrum et al. 2017; Hubbard et al. 2022).

The presence of activity throughout the entire daily cycle suggests that the bobcat exhibits a flexible temporal strategy, although with a tendency to concentrate on activity during crepuscular and nocturnal periods. This strategy is likely associated with optimizing hunting success and synchronizing activity with that of its main prey, particularly lagomorphs and small rodents, which show peaks of activity during crepuscular or nocturnal periods (Tewes et al. 2002; Durán‐Antonio et al. 2020; Draper et al. 2022). In this sense, diurnal activity may be related to opportunistic consumption of prey active during daytime, such as some birds and reptiles, which generally represent a smaller proportion of the species' diet and are considered occasional prey (Litvaitis et al. 1986; Aranda et al. 2002; Tewes et al. 2002; Sánchez‐González et al. 2018; Alanis‐Hernández et al. 2023; Alanis‐Hernández et al. 2026).

Likewise, the greater concentration of activity during low‐light hours may represent a behavioral strategy that reduces the risk of encounters with humans or dominant predators in modified landscapes, as documented for several medium‐sized carnivores (Lendrum et al. 2017; Hubbard et al. 2022). Such temporal adjustments constitute an important adaptive mechanism for species occupying environments subject to varying degrees of anthropogenic disturbance.

Exploratory comparisons between reproductive and nonreproductive periods revealed high temporal overlap in activity patterns (Dhat4 = 0.88; Figure S3), suggesting that the general temporal distribution of bobcat activity remains broadly consistent throughout the year when records are pooled across sampling years. Previous studies have shown that bobcat activity patterns are strongly influenced by prey availability, habitat characteristics, and environmental conditions (Tewes et al. 2002; Lendrum et al. 2017; Shores et al. 2019), factors that help maintain relatively consistent temporal activity throughout the year. Nevertheless, because records were pooled across years and originated from opportunistic observations, subtle seasonal differences may have remained undetected and should be evaluated using standardized long‐term monitoring datasets.

### 5.3 Variation Among Vegetation Types and Behavioral Flexibility

Although the general activity pattern was similar across most analyzed environments, results revealed significant temporal variation among some vegetation types, particularly in comparisons involving Temperate forests (Figure 2). These differences suggest that the bobcat exhibits behavioral adjustments associated with the ecological characteristics of each environment.

In Temperate forests, the observed pattern showed a higher relative proportion of records during the diurnal period compared with other vegetation types, suggesting activity more evenly distributed throughout the day. The relatively higher diurnal activity observed in these environments may be associated with the greater structural complexity and vegetation cover characteristic of Temperate forests, which can reduce predator's visual exposure and allow greater flexibility in activity schedules (Gigliotti et al. 2020). This suggests that responses to anthropogenic disturbance may vary depending on environmental context: in more open or human‐exposed habitats, whereas environments with greater vegetation cover may permit more flexible or partially diurnal activity patterns. Similar patterns have been documented in other mesocarnivores, including coyotes ( *Canis latrans* ), gray foxes ( *Urocyon cinereoargenteus* ), and fishers ( *Pekania pennanti* ), in which diurnal activity may increase in environments with greater vegetation cover that provide more refuge opportunities and lower detection risk (Lesmeister et al. 2015; Lendrum et al. 2017; Parsons et al. 2022). In addition, prey activity in forest environments may show a broader distribution throughout the day, favoring foraging strategies less restricted to crepuscular periods.

In contrast, in Grasslands and Shrublands, activity showed greater temporal concentration around crepuscular periods, consistent with previous studies suggesting that predators in more open environments tend to concentrate activity during lower‐light periods to increase hunting efficiency and reduce exposure (Lendrum et al. 2017; Shores et al. 2019).

In Tropical forests and agricultural‐modified areas, records showed a relatively homogeneous temporal distribution across the daily cycle, with a slightly marked concentration of activity during crepuscular periods. This pattern may indicate greater behavioral flexibility, possibly associated with variable resource availability or the influence of human activities in modified landscapes (Lendrum et al. 2017; Granados et al. 2024).

The pattern observed in riparian vegetation was the most distinctive, characterized by a higher concentration of activity during nocturnal periods. This result should be interpreted with caution due to the small sample size for this vegetation type (*n* = 20). The limited number of records may indicate uneven sampling effort resulting from the opportunistic nature of citizen‐science datasets, including reduced availability of camera traps in riparian environments. Alternatively, bobcats may use these habitats less frequently or primarily as movement corridors in altered habitats and during periods of reduced human activity (Young et al. 2019; Popescu et al. 2021), a pattern documented in other mesocarnivores (Granados et al. 2024). Because our dataset does not allow direct quantification of sampling effort among vegetation types, both explanations should be considered plausible.

Results from the Rayleigh test (Table 3) indicate that in some vegetation types, activity records cluster significantly around a mean temporal direction. In contrast, in other environments, the distribution of records throughout the daily cycle is statistically indistinguishable from a uniform distribution. This pattern suggests that in environments such as Temperate forests, Shrublands, and Grasslands, bobcat activity shows greater temporal aggregation, whereas in Tropical forests, Riparian vegetation, and Agricultural‐modified areas, it may be more diffusely distributed throughout the day.

**TABLE 3:** Results of the Rayleigh test (R) used to evaluate the uniformity of the daily activity pattern of the bobcat ( *Lynx rufus* ) across different vegetation types.

Vegetation	*R*	*p*
Temperate forests	0.10	0.009*
Grasslands	0.19	0.026*
Shrublands	0.25	< 0.001*
Tropical forests	0.10	0.586
Riparian vegetation	0.22	0.396
Agricultural‐modified areas	0.07	0.646

*Note:* Significant departures from circular uniformity are indicated as *.

Rather than reflecting discrete changes in activity type, differences observed among vegetation types appear to respond primarily to variation in temporal concentration of activity throughout the daily cycle. In all analyzed environments, bobcat activity was distributed across 24 h, consistent with a general cathemeral pattern. However, activity density showed shifts in intensity and position of activity peaks among habitats, indicating adjustments in the temporal distribution of behavior rather than categorical changes between diurnal and nocturnal activity. This type of temporal plasticity has been documented in several carnivores whose activity patterns can be redistributed within the daily cycle in response to factors such as prey availability, habitat structure, or human pressure (Frey et al. 2020; Wang et al. 2025).

### 5.4 Comparison With Other North American Mesocarnivores

The temporal flexibility observed for the bobcat is comparable to that reported for other widely distributed mesocarnivores in North America, such as the coyote ( *Canis latrans* ) and gray fox ( *Urocyon cinereoargenteus* ), species that adjust their daily activity in response to factors such as resource availability, habitat structure, and human pressure (Kitchen et al. 2000; Lesmeister et al. 2015; Parsons et al. 2022). However, unlike the coyote, which can increase diurnal activity in highly anthropized environments, the bobcat in most environments tends to concentrate activity during crepuscular or nocturnal periods even in modified landscapes (Figure 2). This difference suggests possible mechanisms of temporal segregation among sympatric carnivores that may facilitate coexistence within communities by reducing direct competition and minimizing overlap in resource use (Neale and Sacks 2001; Riley et al. 2003; George and Crooks 2006; Shores et al. 2019).

### 5.5 Ecological Implications and Future Perspectives

Future research combining citizen science data with standardized monitoring programs will improve the spatial and temporal resolution of these analyses and deepen understanding of the ecological mechanisms that explain variation in activity patterns of carnivores in Mexico.

Our results suggest that the bobcat exhibits substantial temporal plasticity across heterogeneous environments in Mexico, maintaining a broadly cathemeral activity pattern while adjusting the temporal concentration of activity according to habitat characteristics. Such flexibility may represent an important adaptive mechanism that facilitates the persistence of the species across landscapes with contrasting environmental conditions and varying degrees of anthropogenic disturbance.

The study also highlights the potential of citizen science platforms as complementary tools for investigating large‐scale behavioral patterns in widely distributed carnivores. Although opportunistic datasets present important limitations due to uneven sampling effort and habitat representation, integrating rigorous filtering criteria with large volumes of camera‐trap observations can provide valuable ecological insights at spatial scales that are often difficult to achieve through conventional monitoring programs alone.

Future studies integrating citizen science records with standardized camera‐trap networks, environmental variables, and anthropogenic disturbance metrics could improve understanding of the ecological drivers shaping carnivore activity patterns across Mexico. Such approaches may contribute to broader efforts to understand carnivore behavioral responses to landscape transformation and ongoing environmental change.

## Author Contributions

**Luis A. Alanis‐Hernández:** conceptualization (lead), data curation (equal), formal analysis (lead), investigation (lead), methodology (lead), supervision (lead), writing – original draft (equal). **Amayrani E. Trejo‐Montero:** data curation (equal), methodology (equal), writing – review and editing (equal). **Gerardo Sánchez‐Rojas:** writing – review and editing (equal). **Jaime Manuel Calderón‐Patrón:** writing – review and editing (equal). **Mario C. Lavariega:** formal analysis (equal), methodology (equal), validation (equal), writing – review and editing (equal). **Juan Pablo Esparza‐Carlos:** writing – review and editing (equal). **Luis Ignacio Íñiguez‐Dávalos:** writing – review and editing (equal).

## Funding

The authors have nothing to report.

## Conflicts of Interest

The authors declare no conflicts of interest.

## Supporting information


**Figure S1:** Annual number of bobcat ( *Lynx rufus* ) records obtained from iNaturalist camera‐trap observations across Mexico between 2005 and 2025. The number of records increased substantially after 2016, likely reflecting the growing use of citizen‐science platforms and camera‐trap devices. Records for 2025 correspond to a partial year.

**Figure S2:** Monthly distribution of bobcat ( *Lynx rufus* ) records obtained from iNaturalist camera‐trap observations across Mexico between 2005 and 2025. Records were distributed throughout the year but showed variation in monthly frequency, reflecting the uneven temporal distribution characteristic of opportunistic citizen‐science datasets.

**Figure S3:** Daily activity patterns of the bobcat ( *Lynx rufus* ) during reproductive and nonreproductive periods in Mexico based on camera‐trap records obtained from iNaturalist between 2005 and 2025. Solid and dashed lines represent kernel density estimates for reproductive and nonreproductive periods, respectively. Vertical dashed lines indicate periods corresponding to dawn twilight (±1 h before 06:00) and dusk twilight (±1 h before 18:00).

**Dataset: S1** Database of 821 independent camera‐trap records of *Lynx rufus* obtained from iNaturalist in Mexico (2005–2025) and used for activity pattern analyses. (For ease of review, the dataset is attached as an Excel file during the revision process).

## Data Availability

The dataset generated and analyzed during the current study is available as Dataset S1.
