# Supplementary figures and images for: Citizen‐Science Camera Trap Data Reveal Large‐Scale Activity Patterns of the Bobcat (Lynx rufus) Across Mexican Ecosystems
Source: Ecol Evol. 2026 Jul 1;16(7):e73917. doi: 10.1002/ece3.73917 (PMC13322546; doi:10.1002/ece3.73917)

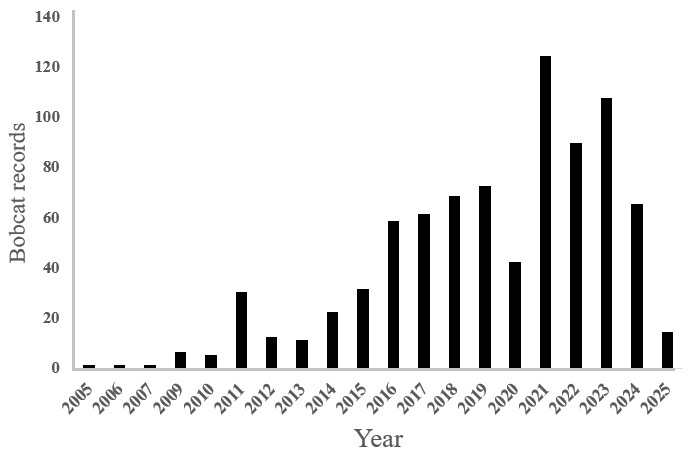

Supplement: Supplementary file 1 — Figure S1: Annual number of bobcat ( Lynx rufus ) records obtained from iNaturalist camera‐trap observations across Mexico between 2005 and 2025. The number of records increased substantially after 2016, likely reflecting the growing use of citizen‐science platforms and camera‐trap devices. Records for 2025 correspond to a partial year. [file ECE3-16-e73917-s001.jpg]

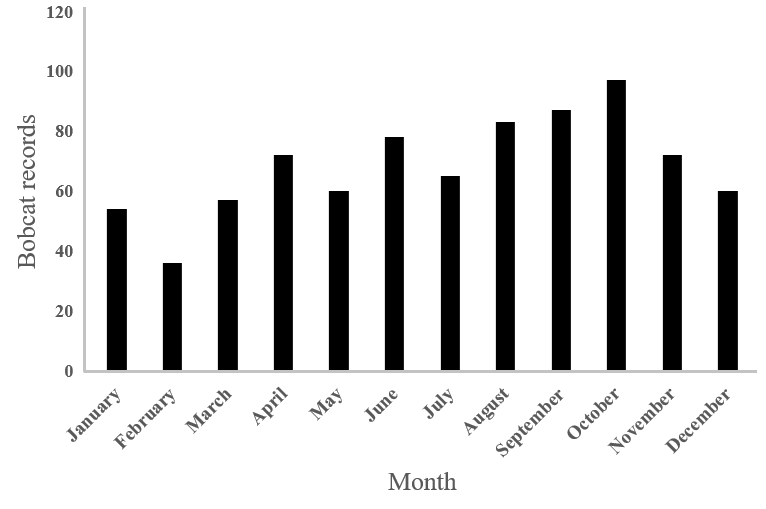

Supplement: Supplementary file 2 — Figure S2: Monthly distribution of bobcat ( Lynx rufus ) records obtained from iNaturalist camera‐trap observations across Mexico between 2005 and 2025. Records were distributed throughout the year but showed variation in monthly frequency, reflecting the uneven temporal distribution characteristic of opportunistic citizen‐science datasets. [file ECE3-16-e73917-s003.png]

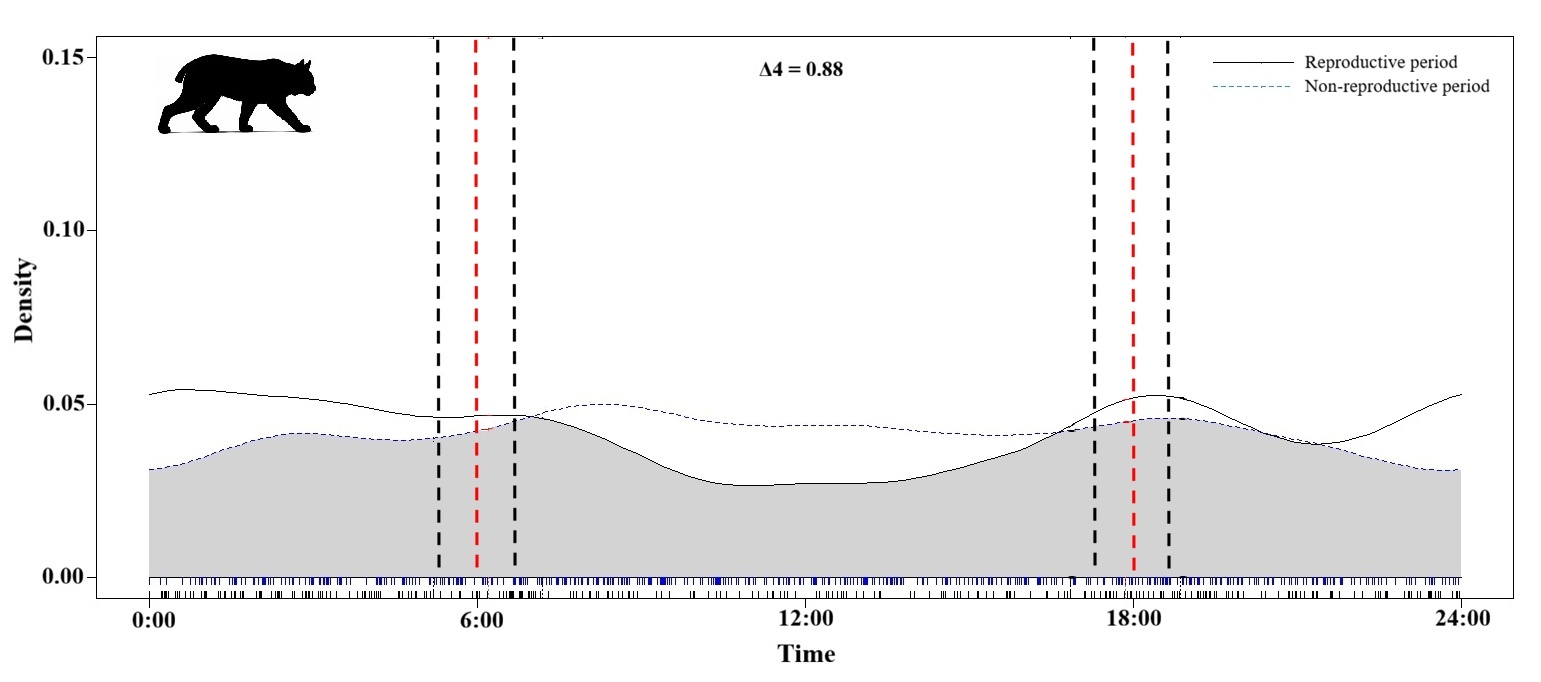

Supplement: Supplementary file 3 — Figure S3: Daily activity patterns of the bobcat ( Lynx rufus ) during reproductive and nonreproductive periods in Mexico based on camera‐trap records obtained from iNaturalist between 2005 and 2025. Solid and dashed lines represent kernel density estimates for reproductive and nonreproductive periods, respectively. Vertical dashed lines indicate periods corresponding to dawn twilight (±1 h before 06:00) and dusk twilight (±1 h before 18:00). [file ECE3-16-e73917-s004.jpg]
